# Supplementary material for: A Computerized Cognitive Test Battery for Detection of Dementia and Mild Cognitive Impairment: Instrument Validation Study
Source: JMIR Aging. 2022 Apr 15;5(2):e36825. doi: 10.2196/36825 (PMC9055476; doi:10.2196/36825)
Supplement: Multimedia Appendix 1 [file aging_v5i2e36825_app1.docx]

Supplementary Information

**Table S1. Cognitive assessments in BrainCheck**

| Assessment | Description | Measurement for Assessment Score | Cognitive Domain |
| --- | --- | --- | --- |
| Immediate and Delayed Recognition | First, Immediate Recognition serially displays 10 words and then asks whether a word was just seen — displays either a distractor word or a target word (20 trials). At the end of the testing battery, without seeing the original list again, participants are again presented with 20 words and asked whether each word was presented before. | Number of correct answers for each assessment | Memory |
| Digit Symbol Substitution | Participants must match an arbitrary correspondence of symbols to digits; when presented with a new symbol, they input the corresponding digit as quickly as possible. | Median duration time of matching the digits and symbols | Executive  function |
| Flanker | Participants are presented with a target item (in this case, a central arrow) flanked by congruent (>> > >>), or incongruent (<< > <<) arrows. Participants identify the direction of the target as quickly and accurately as possible. | Median reaction time of correct direction choice | Alertness,  spatial awareness, and  executive function |
| Stroop | Participants are instructed to find a word matching the given name of a color. There are three types of trials: NEUTRAL in which all words are presented with black font, CONGRUENT in which the word and font color are the same (e.g., the word RED presented in red font), and NON-CONGRUENT in which the word indicates a different color than the font (e.g., the word RED presented in green font). A time-out mechanism is triggered if there is not a completion of a trial in the assessment within 30 seconds. | Median reaction time of incongruent word-color pairs | Executive  function and impulsivity |
| Trail Making Test (Trails) A/B | Participants are instructed to connect a set of 25 dots in their correct order as rapidly as possible. Trail Making Test A uses only numbers (1 through 25), while Trail Making Test B employs alternating numbers and letters (1 – A – 2 – B – 3 – C - …). A time-out mechanism is triggered if there is not a completion of a trial in the assessment within 30 seconds | Median duration of completing the tasks for Trails A and Trails B | Visual  attention and cognitive  flexibility |

_
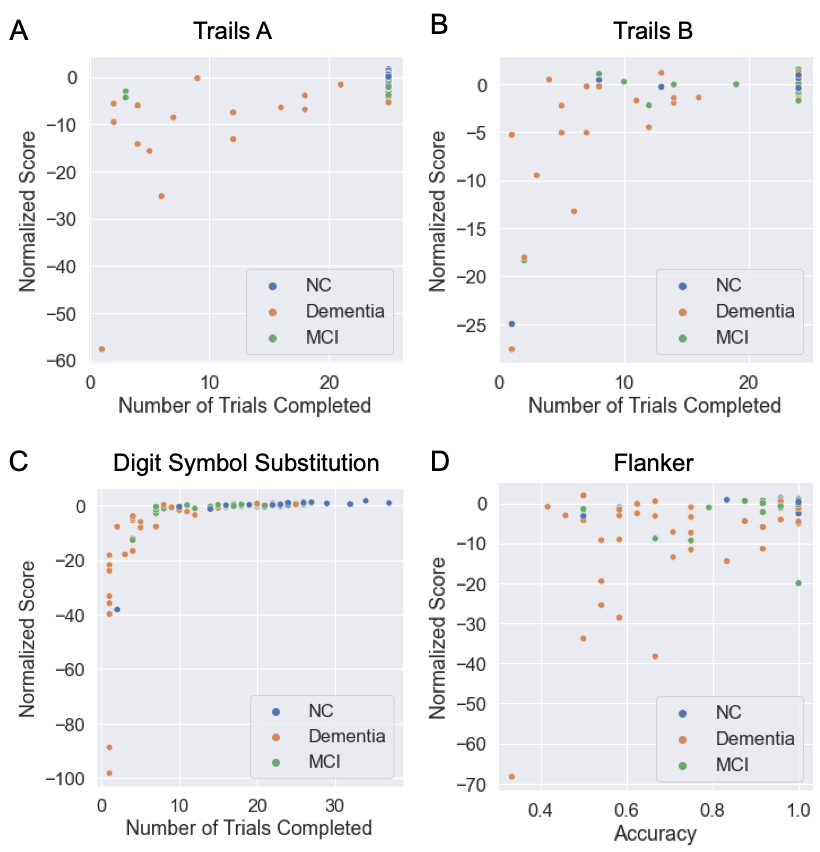
_

**Figure S1. Normalized assessment scores**

(A, B) Trails A/B test showed very low scores if the assessment was not fully completed (time-out). (C) Digit Symbol Substitution showed very low scores if only a few trials were completed in the alloted time. (D) Flanker showed very low scores when the accuracy of the completion was low.
